# Redefining Nutritional Requirements in End-Stage Liver Disease: Towards a Personalized Approach

**DOI:** 10.3390/nu15224770

**Published:** 2023-11-13

**Authors:** Brooke Chapman, Darren Wong, Bethany Whitcher, Marie Sinclair, Paul Gow, Avik Majumdar, Adam Testro

**Affiliations:** 1 Department of Nutrition and Dietetics, Austin Health, Heidelberg, VIC 3084, Australia; 2 Liver Transplant Unit, Austin Health, Heidelberg, VIC 3084, Australia; darren.wong@austin.org.au (D.W.); bethany.whitcher2@austin.org.au (B.W.); marie.sinclair@austin.org.au (M.S.); paul.gow@austin.org.au (P.G.); avik.majumdar@austin.org.au (A.M.); adam.testro@austin.org.au (A.T.); 3 School of Medicine, Dentistry and Health Sciences, The University of Melbourne, Melbourne, VIC 3010, Australia

**Keywords:** indirect calorimetry, cirrhosis, malnutrition, energy requirements, liver transplant

## Abstract

Malnutrition is ubiquitous in cirrhotic patients presenting for liver transplant (LT). Providing an appropriate energy prescription is fundamental to effective nutrition therapy. We aimed to compare measured energy expenditure (mEE) with predicted energy expenditure (pEE) in patients awaiting LT and determine clinical factors associated with mEE. In this prospective observational study, energy expenditure was measured by indirect calorimetry in 110 adult patients referred for LT and predicted by commonly utilized equations (Harris–Benedict, Schofield, and EASL guidelines). Nutritional status, anthropometry, muscle function, biochemical and clinical data were also collected. The median model for end-stage liver disease (MELD) was 19 (IQR 13, 25), and the majority were Child–Pugh B (51%) or C (37%). Malnutrition was evident in 85%. Median mEE by calorimetry was 1756 (1531, 2104) kcal/d and significantly higher than pEE as per Harris–Benedict 1480 (1322, 1722) kcal/d and Schofield 1474 (1349, 1723) kcal/d (both *p* < 0.001), but lower than EASL guidelines (35 kcal/kg) when an activity factor was applied to mEE; 2283 (1990, 2735) kcal/d versus 2590 (2178, 3010) kcal/d (*p* < 0.001). Hypermetabolism (mEE:pEE > 1.2) was evident in 48% of the cohort. Multivariate analysis found MELD, Child–Pugh class, diuretic use, and severe malnutrition to be independent predictors of hypermetabolism. A new liver-specific predictive model has been developed, showing superior agreement with mEE than common predictive equations. In conclusion, there is a poor correlation between mEE and pEE in patients awaiting LTs, and hypermetabolism is common. Relying on historical predictive equations in this patient population may result in significant under or over-feeding. A tailored energy prescription based on indirect calorimetry or a liver-specific predictive model is recommended for LT candidates.

## 1. Introduction

Malnutrition is a frequent complication of liver disease, and its prevalence increases with advancing liver failure. Therefore, patients awaiting liver transplant (LT) have a high incidence of malnutrition [1], which has demonstrated prognostic implications before and after transplant [2,3,4]. Providing an optimal supply of energy (calories) is the cornerstone of nutrition therapy in cirrhosis, where the goal is often to prevent the development or worsening of malnutrition and its associated complications. But whether malnutrition can be reversed in cirrhosis remains contentious. Despite some individual studies demonstrating improved nutritional and clinical parameters [5,6,7,8], multiple meta-analyses have failed to conclude if nutrition therapy improves patient outcomes in liver disease, particularly in relation to survival [9,10,11,12].

The absence of a defined caloric goal, and more importantly, whether this goal is achieved, is a key limitation of many published nutrition studies and could explain the inconsistent benefit observed in the literature to date. When an energy target is provided, it is often based upon a predictive equation (e.g., Harris–Benedict and Schofield). Predictive equations are routinely used in clinical practice to provide a rapid estimation of resting energy expenditure (REE) and utilize a range of patient-specific factors such as age, weight, and height. However, predictive equations are not validated in cirrhosis and are inaccurate when applied to this population [13].

International liver guidelines recommend indirect calorimetry (IC) to measure an individual’s energy requirement in cirrhosis accurately [14,15]. IC measures oxygen consumption (VO_2_) and carbon dioxide production (VCO_2_) and subsequently calculates the daily resting metabolic rate (RMR) for the individual. It provides an accurate and non-invasive measurement of energy expenditure, and various calorimetry machines and techniques exist to obtain measurements in both mechanically ventilated patients (traditional metabolic cart) and spontaneously breathing individuals (portable handheld devices).

However, despite recommendations from best practice guidelines, there is limited data detailing the measured energy expenditure in patients awaiting LT. The application of IC is not yet widespread in hepatology practice or research, and relying on predictive equations to guide nutrition therapy prior to LT poses a significant risk of underfeeding in a patient group that already experiences high rates of malnutrition. IC allows clinicians to personalize the nutrition support prescription to the individual, which is essential if the usual trajectory of worsening malnutrition and deconditioning is to be ameliorated in the pre-transplant period. Therefore, the aim of this study was to compare the measured energy expenditure (mEE) of patients awaiting LT with predicted energy expenditure (pEE) calculated from commonly employed equations and to determine any patient-related or clinical factors associated with mEE.

## 2. Materials and Methods

### 2.1. Patient Population

This prospective observational cohort study was conducted in the Liver Transplant Unit at Austin Health, Melbourne, Australia. Adult patients referred to our center for consideration of liver transplant between March 2019 and July 2022 who had at least one IC measurement undertaken as part of routine clinical care were included. Eligible patients were over 18 years of age, with an indication for LT of decompensated cirrhosis or cirrhosis with hepatocellular carcinoma. Exclusion criteria were as follows: age < 18 years, patients requiring multi-organ transplant, current infection, patients with fulminant liver failure, and patients with hepatic encephalopathy or unable to cooperate. Written informed consent was obtained from all patients, and the study was approved by the Austin Health Human Research Ethics Committee.

The severity of cirrhosis was classified in each patient according to the Child–Pugh model for end-stage liver disease (MELD) scores. Patient demographics (age, gender) and clinical information, including etiology of liver disease, comorbidities, and routine laboratory tests (bilirubin, creatinine, albumin, and INR) were recorded for all patients. The presence and severity of refractory ascites and a history of hepatic encephalopathy were also recorded.

### 2.2. Nutritional Assessment

All patients had weight, height, and body mass index (BMI) recorded before IC measurement. Body weight was measured to the nearest 0.1 kg on calibrated scales. In those with fluid retention, the most recent post-paracentesis weight (if available) was recorded, or dry weight was estimated by subtracting a percentage of weight based on the severity of ascites and the presence of bilateral pedal edema as recommended by European Association for the Study of the Liver (EASL) guidelines [14]. Height was recorded to the nearest 0.1 cm using a stadiometer. The BMI was calculated using the formula weight (kg)/height (m)^2^.

Nutritional assessment was determined by subjective global assessment (SGA) and carried out by specialist LT dietitians, according to Detsky et al. [16]. Patients were classified as well-nourished (SGA-A), mild-moderately malnourished (SGA-B), or severely malnourished (SGA-C). Mid-upper arm circumference was recorded on the left arm at the mid-point between the tip of the acromion process of the shoulder and the olecranon of the elbow to the nearest 0.1 cm. Triceps skinfold (TSF) was measured to the nearest 0.1 mm using skinfold calipers at the anatomical mid-point as described above to measure the fat pad over the left triceps muscle.

Handgrip strength (HGS) was measured with an electronic hand dynamometer (Taeko, TTS^®^, Japan) in the standardized approach described by Roberts [17]. The maximal contraction with the non-dominant arm was recorded to the nearest 0.1 kg.

All measurements were collected by the LT dietitian, who is experienced and trained in measuring anthropometrical markers and muscle strength in this patient group.

### 2.3. Energy Expenditure

Measured energy expenditure (mEE) was obtained by indirect calorimetry using a handheld portable device (Fitmate, Cosmed, Rome, Italy). A ventilated canopy hood was used to measure VO_2_ and calculate VCO_2_. Respiratory gases were then analyzed by the calorimeter and used to calculate REE through the Weir equation [18]. IC measurements were collected by a trained dietitian utilizing standard procedures at our institution to minimize variation, ensuring 10–15 min rest, at least 4 h of fasting, and 12 h of abstinence from exercise prior to IC measurement. All measurements were conducted in a quiet room with patients in a supine position throughout the test.

Prior to each measurement, the calorimeter was calibrated in accordance with the manufacturer’s instructions. The measurement was performed under conditions of absolute rest for up to 20 min, and a steady-state period was selected for analysis, defined as a period of at least 5 min where average VO_2_ and VCO_2_ fluctuated by <10%. mEE was expressed in kcal/d.

To estimate the resting energy expenditure, the Harris–Benedict [19] and Schofield equations [20] were used. Current clinical practice guidelines of the European Association for the Study of the Liver (EASL) recommend a daily energy intake of at least 35 kcal/kg actual body weight per day (in non-obese individuals) [14]; hence, this was also calculated for all patients. To ensure an appropriate comparison of the EASL recommendation (which incorporates an activity factor) with a daily energy target obtained from IC, mEE was multiplied by 1.3 as recommended [14].

### 2.4. Statistical Analysis

Data are presented as the median (IQR), mean (±SD), or frequency and percentage.

Associations between categorical variables were tested with the chi-square test. To compare continuous variables between groups, Student’s *t*-test was used for variables with a normal distribution or the Mann–Whitney *U* test if nonparametric. For intra-group comparisons, the paired *t*-test or Wilcoxon test was used according to their distribution. Correlations were determined using Pearson’s r. Multivariate analysis was performed using multiple linear regression. Predictive modeling was undertaken by training a multiple regression model on 80% of observations. New features were generated for the model by examining patterns of association within the data set. Subsequent model evaluation was performed using the remaining 20%. Agreement between methods of estimating energy expenditure was assessed using the Bland–Altman method. A two-sided *p*-value < 0.05 was considered statistically significant. Statistical analysis was performed using R v4.3.1.

## 3. Results

### 3.1. Patient Characteristics

A total of 110 patients fulfilled the inclusion criteria and were included in the study (Table 1). The majority were male (64%), with a median age of 59 years (IQR 50–64). The leading cause of liver disease was alcohol (33%), followed by non-alcoholic steatohepatitis (NASH) (25%), hepatocellular carcinoma (HCC) (11%), cholestatic diseases (primary biliary cirrhosis and primary sclerosing cholangitis) (11%), hepatitis C virus (HCV) (8%), and other (12%). The severity of liver disease and prevalence of decompensation symptoms were reflective of a cohort referred for LT. Median MELD was 19 [13,14,15,16,17,18,19,20,21,22,23,24,25], with more than one-third of patients (34%) having a MELD between 21–30 and 8% with a MELD greater than 30. The majority were Child–Pugh B (51%) or C (37%). Ascites was present in 60%, whilst 47% had a history of hepatic encephalopathy.

Prevalence of malnutrition was also high, with 85% of patients malnourished as diagnosed by SGA (48% mild-moderately malnourished; 37% severely malnourished), despite median BMI at the upper end of the healthy weight range. Median dry weight was 74 kg (62–86). Median muscle strength was below sarcopenic cut-off values in both men and women [21].

### 3.2. Difference between Measured Resting Energy Expenditure and Estimation of Energy Requirements

**Figure 1:**
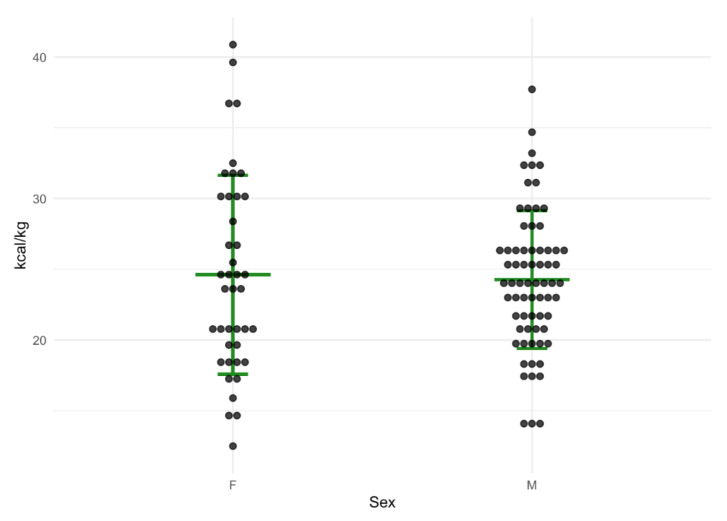
Dot plot of resting energy expenditure in kcal/kg, as measured by indirect calorimetry in 110 patients, separated by gender (male *n* = 69, female *n* = 41). Horizontal green lines indicate median and interquartile ranges.

The group median resting energy expenditure measured by IC was 1756 kcal/d (1531–2104), equivalent to 24.4 kcal/kg/d when considering the median weight of patients, though ranged from 12.5 kcal/kg to 41 kcal/kg for individual patients (Figure 1).

Table 2 shows the energy requirements of patients as determined by different methods, both measured (IC) and predicted (equations). IC measurements were significantly higher than that estimated by both the Harris–Benedict (1480 kcal/d (1322–1722)) and Schofield (1474 kcal/d (1349–1723)) equations, with a mean adjusted difference (MAD) of 271.03 and 260.81 kcal/d, respectively (both *p* < 0.001) (Table 3). The ratio of mEE to pEE ranged from 56% to 202%, and only 23.6% of patients (*n* = 26) had a mEE within the accepted limits of 90 and 110% of pEE as determined by Harris–Benedict and Schofield equations. Most patients (*n* = 71, 64.6%) had a mEE greater than 110% of the pEE, whilst 13 patients (11.8%) had a mEE below 90% of the pEE.

When comparing IC with the EASL guidelines (35 kcal/kg), which are a recommendation for energy supply to balance total energy expenditure (incorporating not only REE but also food-related thermogenesis and energy expenditure related to physical activity), the IC value was multiplied by 1.3 as per the recommended standard convention (14). The median predicted EE with the EASL equation was 2590 kcal/d (2178–3010), and significantly higher than measured EE with a 1.3 multiplication factor applied of 2283 kcal/d (1990–2735) (*p* < 0.001). The MAD of adjusted IC with the EASL recommendation was −297.92 kcal/d (*p* < 0.001) (Table 3).

Absolute resting energy expenditure was significantly higher in men than women regardless of the method used (*p* all < 0.001) (Table 2); however, this was primarily attributed to body weight. There was no difference in individual energy expenditure between men and women when weight was taken into account and expressed as kcal/kg (*p* = NS).

### 3.3. Correlation between Measured and Predicted Energy Expenditure

**Figure 2:**
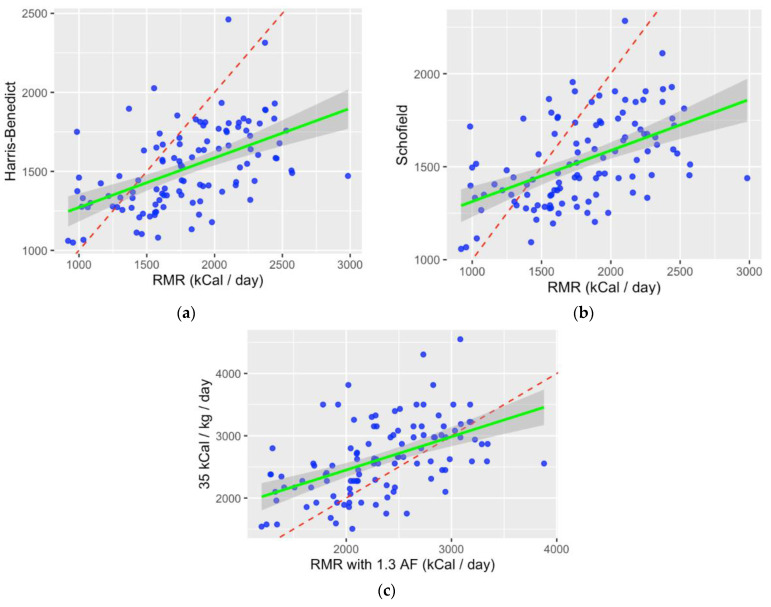
Pearson correlation scatter plots with fit. Red dashed lines indicate measured RMR via indirect calorimetry. Blue dots represent individual prediction via method of estimation. Green solid line indicates line of best fit. (**a**) Correlation of measured RMR (kcal/d) via indirect calorimetry with Harris–Benedict equation; (**b**) Correlation of measured RMR (kcal/d) via indirect calorimetry with Schofield equation; (**c**) Correlation of measured RMR (kcal/d), adjusted for activity (×1.3) with 35 kcal/kg EASL recommendation.

When comparing indirect calorimetry with the estimated REE by prediction equations, there was poor correlation. Scatter plots illustrate that Harris–Benedict (Figure 2a) and Schofield (Figure 2b) both underestimate energy requirements; EASL (Figure 2c) overestimates energy requirements.

### 3.4. Parameters Associated with Hypermetabolism

Hypermetabolism was defined as a mEE:pEE ratio greater than or equal to 1.2. A total of 53 patients (48%) exceeded 120% of their pEE and thus were classified as hypermetabolic. There was no difference in gender distribution between hypermetabolic and normometabolic patients. Hypermetabolic patients were more likely to have more advanced liver disease as per the MELD/MELD-Na score, have refractory ascites, and be severely malnourished (Table 4). Female patients exhibiting hypermetabolism had poorer anthropometric indices, with males demonstrating a similar, though non-significant, trend.

A multivariate analysis was then performed to identify factors independently related to increased energy expenditure of patients awaiting liver transplants, with hypermetabolism as a dependent variable. Significant factors from the univariate analysis were included as potential independent variables. Only MELD [odds ratio (OR): 1.08, 95% confidence interval (CI): 1.02–1.14; *p* = 0.008], MELD-Na (OR: 1.07, 95% CI: 1.02–1.13; *p* = 0.005), Child–Pugh class (OR: 1.25, 95% CI: 1.02–1.54; *p* = 0.032), severe malnutrition (OR: 7.51, 95% CI: 2.04–36.8; *p* = 0.005), and use of diuretics (OR 2.81, 95% CI: 1.14–7.49; *p* = 0.03) were found to be independently related to the presence of hypermetabolism (Table 5).

### 3.5. Development of a New Prediction Model

**Figure 3:**
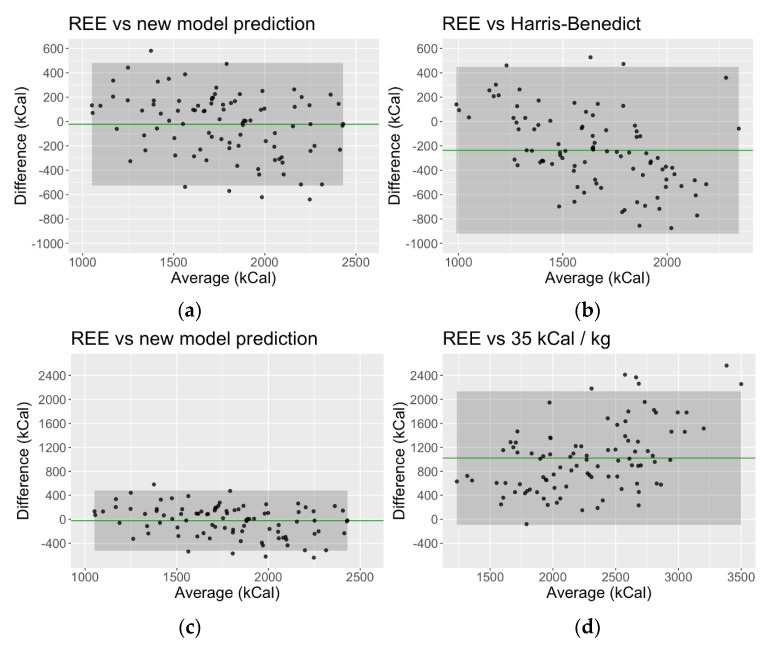
Bland–Altman concordance plots showing limits of agreement for mEE by IC versus pEE by the new liver-specific model, HB and EASL equations. Green solid line in each plot indicates mean difference (bias), and shaded area shows 95% confidence interval. (**a**) REE vs. new model prediction. The bias (mean difference) between mEE and the new model is −22.14 kcal [95% CI: −524.14, 479.85]. The concordance correlation coefficient is 0.77; (**b**) REE vs. Harris–Benedict. The bias between mEE and HB equation is −235.78 kcal [95% CI: −920.11, 448.56]. The concordance correlation coefficient is 0.41; (**c**) REE vs. new model prediction, as above, shown in scale comparison to (**d**); (**d**) REE vs. EASL. The bias between mEE and EASL equation is 1021.02 kcal [95% CI: −93.93, 2135.98]. The concordance correlation coefficient is 0.18.

Given the problems encountered with the commonly utilized prediction equations identified above, a new liver-specific predictive equation would be beneficial to clinicians. To develop this model, an examination of these data and their associations with mEE identified that dry weight (kg), Child–Pugh category, handgrip strength (kg), age (years), and SGA class C were strongly predictive. New features identified included converting MELD into categories (≤15, 16–20, 21–30, ≥31) as well as age (years) squared and an additional factor if age was greater than 65 years. There was also an interaction between dry weight and SGA class C. These measured variables and new features were significant in the new model, accounting for approximately 63% of the observed variance in mEE. Importantly, the average degree of underestimation in calorie requirements was markedly reduced by the new predictive model, with superior agreement between mEE by calorimetry and the new predictive model compared to other predictive equations (Figure 3).

The new liver-specific prediction equation is as follows, with additional factors presented in Table 6 to add to the result, where applicable:

$$RMR=2683.9\;+\;5.8\times dry\;weight+21.0\times HGS-24.9\times age\;-51778.5\times age^{2}$$

## 4. Discussion

In this study of 110 patients with advanced cirrhosis awaiting liver transplantation, predicted energy expenditure, calculated using three equations commonly employed in clinical practice, was compared with measured energy expenditure, determined by indirect calorimetry. We found that the Harris–Benedict and Schofield equations significantly underestimated energy requirements, and EASL recommendations overestimated energy requirements for patients awaiting LT. Our findings demonstrate significant individual variation in measured requirements and confirm that current predictive equations should not be considered a definitive guide for prescribing nutrition interventions in cirrhotic patients awaiting transplant.

Determining a precise caloric target for patients with advanced liver disease is critical. As our data confirms, the prevalence of malnutrition in this patient population is high, and poor nutritional status is established as a key contributor to the development of sarcopenia [22]. The impact of malnutrition and sarcopenia on pre- and post-transplant outcomes is well known [14,15,23]. Increasingly, the importance of declining nutritional status and muscle strength is also being recognized [1,24,25,26], and if the trajectory of nutritional and functional decline is to be reversed, an adequate energy prescription needs to be established and achieved.

The poor agreement we observed between IC and predictive equations is consistent with other studies in liver disease. Underestimation of energy requirements, as determined by Harris–Benedict, has been reported in populations with a similar clinical acuity to ours [27,28,29,30], with a mean difference in measured to predicted requirements of 311 kcal in one study [28], which is even greater than the 271 kcal discrepancy that we observed. This under-prediction of energy expenditure is concerning not only because of the magnitude of the difference but in particular given that it was largely observed in a malnourished and high-risk group of patients (SGA B and C). Conversely, some studies have found mEE to be lower than pEE as determined by Harris–Benedict in patients with liver disease [31,32]. However, common features of these data are from populations with a lesser severity of liver disease (lower MELD score, greater proportion of Child–Pugh A), and lower prevalence of malnutrition when compared to our population.

In addition to the propensity for predictive equations to underestimate energy requirements in pre-transplant patients, there was a high degree of variability at an individual level. An acceptable range of error (±10%) is commonly utilized when comparing mEE and pEE [33]. Less than a quarter of our population (*n* = 26) had a measured metabolic rate within the accepted range of Harris–Benedict and Schofield equations. Similarly, in a study of cirrhotic patients with ascites where RMR was measured by calorimetry at baseline and four weeks after large-volume paracentesis, only 37% of energy estimations were within 10% of mEE at baseline, which reduced to 26% at four weeks after paracentesis [34]. A meta-analysis of 17 studies (*n* = 1883) demonstrated greater agreement between predicted versus measured energy expenditure in cirrhosis than our study, though it still found only 45% of predictive equations were within 10% of measured RMR by IC [35].

The degree of hypermetabolism in our study was 48%, which is within the prevalence range of 5.3 to 58.3% hypermetabolism reported in a recent literature review of cirrhotic patients [13]. The high prevalence of hypermetabolism in our cohort is a key factor contributing to the inconsistency between mEE and pEE we observed. There are currently no accepted clinical or biochemical factors that can predict hypermetabolism in liver disease. Like our study, some researchers have found hypermetabolism to be significantly associated with the severity of liver disease [36,37]; however, this is not always the case. Many agree that extrahepatic elements of cirrhosis are major determinants driving increased metabolic rate. Factors independently associated with hypermetabolism include elevated fasting glucose [28], adiponectin [38], insulin resistance [27], and higher fat-free mass [27,38]; with conflicting data regarding high [39] and low levels of leptin [27,39]. The mechanisms underlying these factors and hypermetabolism are not precisely understood. Further exploration should be carried out to discover the role of these factors in increasing metabolism in liver cirrhosis. It is worth noting we had 13 patients (11.8%) with hypometabolism, where mEE was >10% lower than the Harris–Benedict and Schofield equations. Small numbers precluded any meaningful analysis of hypometabolism but further highlighted the importance of individual measurement of energy expenditure in patients with cirrhosis to prevent both under and overfeeding.

These data highlight the lack of clinical utility of current predictive equations in patients with cirrhosis. Best practice guidelines, therefore, support the use of IC to guide nutrition prescription in liver disease [14,15], but the application of IC in clinical practice remains limited due to the level of resourcing required to undertake IC measurements routinely. Where calorimetry is not available or inappropriate, predictive equations are used to guide the energy prescription. Although many predictive equations for resting energy expenditure exist, none have been developed for a population with advanced liver disease. The most widely used equation, Harris–Benedict, was developed over 100 years ago from healthy, lean (mean BMI 21.5 kg/m^2^), and predominantly young (average age 30 years) males. Patients with end-stage liver disease awaiting transplant have a vastly different profile. Our cohort was significantly older, heavier, and sicker than the population this equation was derived from; hence, it accounts for much of the discrepancy we observed between measured and predicted energy expenditure. Herein, we have developed a new equation, which is specific for a population with advanced cirrhosis, and advocate for its use in this setting. In comparison to commonly used prediction equations, we have developed a new model that appears to provide a more accurate estimation of energy requirements, with superior concordance to measured energy expenditure than both Harris–Benedict and EASL equations. In addition to improved accuracy, the new model includes liver-specific parameters that are easily obtainable by the clinician, including the MELD score, Child–Pugh category, nutritional status, and handgrip strength. These readily available measures will enhance the practical application of the new model. Other authors have also attempted to develop a model for predicting nutrition requirements for patients undergoing liver transplant. In a study of 143 adult patients after LT, Lindqvist et al. found age, sex, body weight, operative time, MELD, cold ischaemic time, and steroid dose were significant contributors to REE and accounted for 42% of the variance of mEE [40]. In comparison, our model accounts for 63% of the variability in mEE, with narrower limits of agreement −524.14 to +479.85 kcal, compared to −682 to +686 kcal for their model [40].

Our study was conducted in a niche patient cohort in a single center; hence, this poses a potential limitation. To overcome this, we have attempted to provide as much detail as possible about the population so that readers can determine the applicability of results to their own cohort. We also acknowledge that a single IC measurement does not account for the variation inherent in patients with advanced liver disease, particularly as complications of cirrhosis arise, and that repeated IC measures over time are recommended to monitor patient progress and response to nutrition interventions. A multicenter prospective study would address these limitations and enable the evaluation of the impact of targeted nutrition prescribing on clinical and nutritional outcomes. Although the sample size was relatively large for a study in this specific population, it may have been underpowered to uncover other significant findings, particularly in relation to hypermetabolism. Finally, our proposed new model requires external and prospective validation.

## 5. Conclusions

In conclusion, this study showed there is poor agreement on both a group and individual level between EE measured by IC and energy requirements calculated using equations. We suggest that the Harris–Benedict, Schofield, and EASL equations should not be used to estimate REE in cirrhotic patients awaiting liver transplant. Individual measurement of energy expenditure is the preferred method, and newer, portable calorimeters, as utilized in this study, offer broader clinical utility compared to historic metabolic carts. In the absence of IC, an alternative, liver-specific equation has been developed that showed greater agreement with IC than the aforementioned predictive formulae, and this requires validation in other cirrhotic cohorts. Further studies are required to assess whether targeted nutrition therapy provides nutrition or clinical benefits for patients awaiting LT.

## Figures and Tables

**Table 1:** Demographic and clinical characteristics of patients.

Characteristic	Total (*n* = 110)
Male, *n* (%)	70 (64)
Age (years), median (IQR)	59 (50–64)
[Age range]	(21–71)
Aetiology:	
Alcohol	36 (33)
NASH	28 (25)
HCC	12 (11)
Cholestatic disease	12 (11)
HCV	9 (8)
Other	13 (12)
MELD, median (IQR)	19 (13–25)
MELD-Na, median (IQR)	21 (14–28)
Child–Pugh Score, median (IQR)	9 (8–10)
Child–Pugh Category, *n* (%)	
A	13 (12)
B	56 (51)
C	41 (37)
Ascites, *n* (%)	65 (60)
History hepatic encephalopathy, *n* (%)	52 (47)
Weight (kg), median (IQR)	74 (62–86)
BMI (kg/m^2^), median (IQR)	25.2 (22.2–28.4)
Nutritional status, *n* (%)	
SGA-A (well nourished)	16 (15)
SGA-B (mild-moderately malnourished)	53 (48)
SGA-C (severely malnourished)	41 (37)
HGS (kg), median (IQR)	
Male	26.3 (22.6–31.6)
Female	17.1 (14.4–22.4)
MUAC (cm), median (IQR)	
Male	29 (25.5–31.7)
Female	26.7 (23.7–32.1)
TSF (mm), median (IQR)	
Male	11.6 (8.4–15)
Female	11.0 (7.9–16.9)

IQR, interquartile range; NASH, non-alcoholic steatohepatitis; HCC, hepatocellular carcinoma, HCV, hepatitis C virus; MELD, model for end-stage liver disease; BMI, body mass index; SGA, subjective global assessment; HGS, handgrip strength; MUAC, mid-upper arm circumference; TSF, triceps skinfold.

**Table 2:** Resting energy expenditure (kcal/d) obtained by different methods.

Method	Total (*n* = 110) Median	IQR	Female (*n* = 41) Median	IQR	Male (*n* = 69) Median	IQR
REE IC	1756	1531–2104	1583	1305–1755	1926	1616–2229
REE H-B	1480	1322–1722	1318	1256–1424	1638	1435–1800
REE Schofield	1474	1349–1723	1344	1276–1414	1641	1454–1766
REE IC×1.3	2283	1990–2735	2058	1697–2282	2504	2100–2898
REE EASL	2590	2178–3010	2275	1925–2555	2800	2468–3150

REE, resting energy expenditure; IC, indirect calorimetry; H-B, Harris-Benedict; EASL, European Association for the Study of the Liver.

**Table 3:** Comparison between prediction formula and IC in the assessment of resting energy expenditure.

Method	Mean Adjusted Difference	*p*
IC with Harris–Benedict	271.03	<0.001
IC with Schofield	260.81	<0.001
IC×1.3 with EASL	−297.92	<0.001

IC, indirect calorimetry; EASL, European Association for the Study of the Liver.

**Table 4:** Comparison of nutritional and clinical factors between hypermetabolic and normometabolic patients awaiting liver transplant.

	Normometabolic (*n* = 57)	Hypermetabolic (*n* = 53)	*p*
Male, *n* (%)	37 (65)	33 (62)	NS
MELD, median (IQR)	17 (12–23)	20 (16–28)	0.003
MELD-Na, median (IQR)	20 (13–23)	24 (16–31)	0.002
Refractory Ascites, *n* (%)	30 (52)	34 (64)	0.04
Hepatic Encephalopathy, *n* (%)	27 (47)	25 (47)	0.46
Weight (kg), median (IQR)	75 (65–90)	74 (60–85)	0.14
Well-nourished, *n* (%)	13 (23)	3 (6)	
Moderately malnourished, *n* (%)	29 (51)	24 (45)	
Severely malnourished, *n* (%)	15 (26)	26 (49)	0.01
HGS (kg), median (IQR)			
Male	26.7 (22.7–33.6)	25.9 (22.5–30.3)	0.18
Female	16.95 (14.3–22.7)	17.1 (14.8–20.5)	0.47
MUAC (cm), median (IQR)			
Male	29.2 (26.5–32.7)	28.3 (25–30.7)	0.15
Female	28 (26.5–32.5)	23.7 (21.4–26.9)	0.03
TSF (mm), median (IQR)			
Male	12.2 (8.6–17.7)	10.4 (8.2–13.7)	0.08
Female	13 (10.8–19.1)	8.0 (6.4–12.8)	0.02

IQR, interquartile range; MELD, model for end-stage liver disease; HGS, handgrip strength; MUAC, mid-upper arm circumference; TSF, triceps skinfold.

**Table 5:** Multiple linear regression of independent factors for hypermetabolism.

Characteristic	*n*	OR	95% CI	*p*
Diuretics	110	2.81	1.14, 7.49	0.03
MELD	110	1.08	1.02, 1.14	0.008
MELD-Na	110	1.07	1.02, 1.13	0.005
Child–Pugh score	110	1.25	1.02, 1.54	0.032
Nutritional Status:				
SGA A	13	-	-	
SGA B	56	3.59	1.01, 17.0	0.067
SGA C	41	7.51	2.04, 36.8	0.005

MELD, model for end-stage liver disease; SGA, subjective global assessment; OR, odds ratio; CI, confidence interval.

**Table 6:** Additional factors for liver-specific prediction equation.

Modifier	Change to Estimate
Child–Pugh Category	
B	+281.3
C	+390.4
MELD Score	
16–20	+108.5
21–30	+250.1
>31	+292.7
If age >65 years	+73.9
If SGA-C	+15.3 × *dry weight* − 956.2

## Data Availability

Data is contained within the article.
